# Determining the impact of cannabis use and severity on tobacco cessation outcomes: study protocol for a prospective tobacco treatment trial

**DOI:** 10.1186/s40359-023-01060-2

**Published:** 2023-01-25

**Authors:** Kyle J. Walters, Nathaniel L. Baker, Rachel L. Tomko, Kevin M. Gray, Matthew J. Carpenter, Erin A. McClure

**Affiliations:** 1 Department of Psychiatry and Behavioral Sciences, Medical University of South Carolina, 67 President St., MSC 861, Charleston, SC 29425 USA; 2 Department of Public Health Sciences, Medical University of South Carolina, Charleston, SC 29425 USA; 3 Hollings Cancer Center, Medical University of South Carolina, 86 Jonathan Lucas St., Charleston, SC 29425 USA

**Keywords:** Tobacco cessation, Treatment, Cannabis, Marijuana, Varenicline, Co-use, Polysubstance use, Pharmacotherapy

## Abstract

**Background:**

Several evidence-based tobacco cessation treatment strategies exist, though significant barriers to cessation remain which must be addressed to improve abstinence rates for sub-populations of those smoking cigarettes. Cannabis co-use among those who use tobacco is common and appears to be increasing among adults in the United States (US). The literature evaluating the impact of cannabis use on tobacco cessation has been mixed and has several important limitations, which precludes development of treatment recommendations specific to individuals who use tobacco and co-use cannabis. To date, no prospective studies have evaluated the impact of cannabis use and severity on tobacco cessation or quantified cannabis use changes during tobacco treatment to assess for concurrent reductions, abstinence, or compensatory (i.e., increased) cannabis use. This study’s aims are to: (1) evaluate tobacco cessation outcomes among participants who co-use cannabis compared to participants only using tobacco, (2) using daily diaries and biochemical verification, assess changes in cannabis use during tobacco treatment, and (3) assess for a dose-dependent impact of cannabis use on tobacco cessation.

**Method:**

A multi-site, prospective, quasi-experimental 12-week tobacco treatment trial enrolling treatment-seeking adults (ages 18–40; N = 208) from three sites across South Carolina (US) who use tobacco daily and oversampling (2:1) those who co-use cannabis. Participants receive tobacco cessation pharmacotherapy (varenicline) paired with behavioral support, while cannabis use is not addressed as part of treatment. The primary outcome is 7-day point prevalence tobacco abstinence at the week 12 end of treatment visit, measured via biochemical verification and self-report. Secondary outcome measures include changes in cannabis use (via biochemical verification and self-report) during tobacco cessation treatment.

**Discussion:**

Results from this trial have the potential to inform tobacco treatment among those co-using cannabis, which may require a tailored approach to address the role of cannabis in quitting tobacco.

***Trial registration*:**

The trial is registered with ClinicalTrials.gov: NCT04228965. January 14th, 2020.

## Introduction

Combustible tobacco use (mostly via cigarette smoking) in the United States (US) has seen a continual decline over the past several decades [1–3], though the resulting health burden from tobacco continues to be significant and remains the leading cause of preventable death [4]. Disparities exist among those using tobacco, particularly among individuals with co-occurring substance use. Rates of tobacco use are two to three times higher in those with co-occurring substance use disorders compared to the general population [5–7]. The co-use of cannabis in particular is common and rates of cannabis co-use appear to be rising. Rates of daily cannabis use among those smoking cigarettes daily nearly doubled between 2002 and 2014 [8], and cannabis use prevalence has been consistently higher among those who smoke cigarettes compared to those who do not smoke [9]. Recent estimates suggest that 24–29% of those smoking cigarettes in the US co-used cannabis in the past 12 months and 35% of those smoking cigarettes daily also used cannabis daily [10, 11]. Co-use refers broadly to the use of both substances within an individual but varies in temporal proximity and relatedness. For example, tobacco and cannabis can be used sequentially (e.g., cannabis use followed by tobacco use), co-administered (i.e., simultaneous use in the same preparation), or in an independent manner [12]. Harms associated with co-use have been documented, including psychiatric and psychosocial problems [13, 14], health-related problems, and increased toxicant exposure [15–19]. Further, cannabis-tobacco co-use may adversely influence tobacco treatment.

The literature assessing the impact of co-use on treatment outcomes has been mixed. One systematic review found no clear effect of co-use on tobacco cessation [20]. In a separate review of treatment-related studies, we found mixed results regarding adverse impacts of co-use on tobacco use (N = 9 showing an adverse impact; N = 7 showing no impact) [21]. Since that review, three additional papers have been published that have also shown mixed findings on the impact of co-use on tobacco cessation [22–24]. This literature has notable limitations, including methodological variation and varying study samples (secondary data analyses, national surveys), lack of biochemical verification of cannabis use, and sample heterogeneity [21]. To date, no prospective studies have been conducted to evaluate the impact of cannabis use and severity on tobacco cessation, thus limiting our ability to inform treatment recommendations specifically designed for those co-using cannabis and tobacco.

Another treatment-related concern among those who are co-using cannabis involves the potential for compensatory (increased) use of cannabis during tobacco treatment. Studies have either shown no differences in cannabis use during tobacco treatment or have demonstrated a reduction or concurrent decreases in cannabis use [25, 26]. In a cross-sectional online survey of those who self-reported co-use, 50% of survey respondents retrospectively reported increases in cannabis use during a past tobacco quit attempt [27]. Notably, many tobacco cessation trials either exclude for cannabis co-use meeting clinical thresholds for cannabis use disorder or do not capture granular cannabis use data to sufficiently evaluate changes in use patterns [12].

With recent increases in the prevalence of tobacco and cannabis co-use among adults and the potential treatment implications of co-use, a better understanding of the relationship between cannabis use and its severity on tobacco cessation is needed. This trial protocol describes an ongoing prospective tobacco treatment trial in which adults who smoke cigarettes daily and are interested in quitting are enrolled (ages 18–40; N = 208), and participants who co-use cannabis are oversampled 2:1. This study has the following aims: (1) examine the impact of cannabis co-use on tobacco cessation among participants who are co-using compared to participants only using tobacco in a 12-week tobacco cessation trial; (2) using daily diaries and biochemical verification, assess changes in cannabis use (among those co-using cannabis) during tobacco treatment; and (3) assess for a dose-dependent impact of cannabis use on tobacco cessation outcomes.

## Methods

### Research design

This ongoing multi-site study is a prospective 12-week tobacco cessation trial (Clinical Trials ID: NCT 04228965) but is specifically enrolling and oversampling (2:1) those who co-use tobacco and cannabis. All participants (ages 18–40; N = 208) receive first-line tobacco cessation pharmacotherapy (varenicline), in addition to behavioral support (abstinence-based contingency management and cessation counseling) over the 12-week treatment period. Biochemical indices of both tobacco and cannabis use are collected throughout the 12-week treatment, in addition to self-reported use through mobile daily diaries. Cannabis use is assessed throughout the study but is not addressed as part of treatment. The goal of this study is not to evaluate the tobacco cessation treatment being implemented, but rather, tobacco treatment is being used to promote abstinence and test hypotheses regarding tobacco cessation among those who co-use cannabis. All study procedures have been approved by the Institutional Review Boards (IRB) of the Medical University of South Carolina (MUSC). Enrollment is expected to conclude in December 2023, with final primary outcome collection in March 2024.

### Participants

This study is being conducted at three sites across South Carolina. Participants are recruited from the community and must meet the following inclusion criteria: (1) ages 18–40; (2) daily cigarette smoker for ≥ 6 months, smoking ≥ 5 cigarettes per day; (3) must submit a breath carbon monoxide (CO) sample of ≥ 7 parts per million at screening; (4) interest in quitting smoking cigarettes (5+ on a 10-point scale); and 5) must be willing to take varenicline during the 12-week study. Additional inclusion criteria for the cannabis co-using sample include: (1) use of cannabis on at least 10 out of the past 30 days; and/or (2) positive qualitative urinary cannabinoid test at screening (limit of detection is 50 ng/ml). Quit interest for cannabis is not a requirement for inclusion. To be categorized as a tobacco-only control, participants are required to submit a negative qualitative urinary cannabinoid test at screening and self-report fewer than 10 days of cannabis use in the past 30 days.

Participants are excluded from study participation under the following conditions: (1) any serious or unstable medical/psychiatric disorder (including severe substance use disorders, other than cannabis use disorder [CUD]) in the past 3 months that may interfere with study performance or affect safety; (2) currently pregnant or breastfeeding; (3) current use of medications with tobacco cessation efficacy; (4) use of any medications that would interfere with varenicline; or (5) regular use of tobacco or nicotine products other than combustible cigarettes. We do not exclude participants if they are using non-combustible methods of cannabis (e.g., wax, dabs, etc.).

### Procedures

**Fig. 1:**
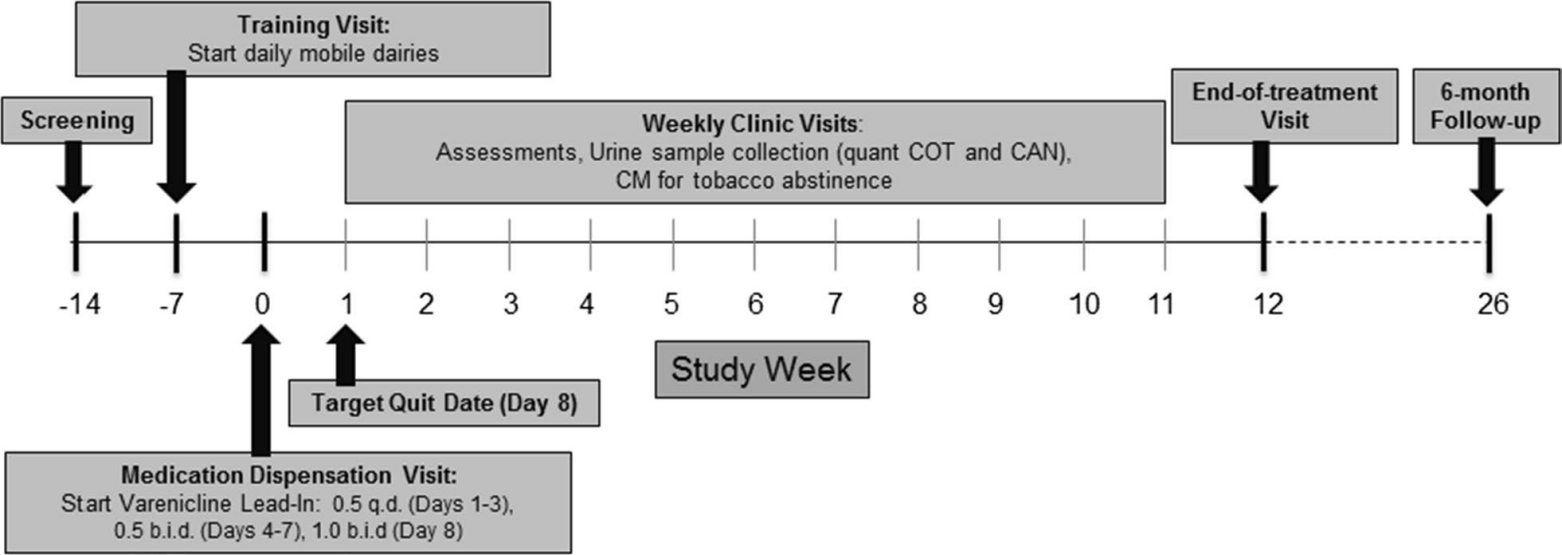
Study design

The study design is shown in Fig. 1. Following a prescreening to determine initial eligibility, participants complete informed consent and screening/baseline assessments. Prior to enrollment and medication initiation (Day 0), participants complete a training visit, where they are instructed how to record and upload medication videos twice daily through the Research Electronic Data Capture (REDCap) platform [28] to assess adherence throughout the trial. On Day 0, participants are given study medication (varenicline) and counseled regarding their upcoming tobacco quit attempt (Day 8). Participants then attend weekly clinic visits until the week 12 end of treatment (EOT) visit. Weekly study visits include the collection of adverse events, urine and breath sample collection, brief smoking cessation counseling, and completion of self-report assessments. Participants then return for post-treatment follow-up visits at weeks 16 (remote only) and 26 (in-person). Participants may earn a total of $1050 for study participation ($530 for study visits, $220 for biologically-confirmed tobacco abstinence at weekly visits, and $300 for completion of daily diaries).

### Study interventions

#### Varenicline

Varenicline is administered to all study participants during the 12-week treatment phase. Dosing is consistent with the package insert and includes 0.5 mg once per day (q.d.) on Days 1–3, 0.5 mg twice per day (b.i.d.) on Days 4–7, and 1.0 mg b.i.d. starting on Day 8 and lasting until the week 12 EOT visit. If participants experience medication-related adverse events, they meet with a study medical clinician and potentially undergo a temporary or sustained dose reduction. If dose reductions do not improve adverse events, medication may be discontinued, but participants are retained in the study.

#### Contingency management

All participants receive financial incentives based on biochemical verification of abstinence from tobacco during weekly visits. A set amount of $20 per sample (collected at each weekly visit) is delivered based on a negative qualitative urinary cotinine result (cut-off of 200 ng/ml). If remote sessions are conducted, participants are provided with take-home urine supplies to complete their instant-read test through video chats with research staff to verify results and provide incentives, if negative.

#### Counseling

Participants engage in psychosocial counseling at Day 0 in preparation for the quit attempt and throughout treatment at weekly study visits. Counseling is brief (~ 5 to 10 min per session) and consists of understanding triggers, dealing with cravings, adjusting to a smoke-free lifestyle, cognitive restructuring, changing routines, and how to manage lapses. Trained research staff deliver counseling using a guide developed for topics to cover each week.

### Primary and secondary outcome measures

The primary outcome is 7-day point prevalence tobacco abstinence (PPA) at the week 12 EOT study visit, measured via biochemical verification and self-report. The secondary outcome is changes in cannabis use during tobacco cessation treatment. Among participants co-using cannabis, cannabis use frequency and amounts during the final 4 weeks of tobacco treatment (weeks 8–12) will be assessed and nicotine dependence at baseline will be the primary model predictor. Finally, we will assess cannabis co-use severity on tobacco cessation outcomes as an exploratory aim.

### Measures/assessments

#### Biochemical measures of substance use

Biochemical verification of tobacco, cannabis and other substances is completed at all study visits. Urine samples are collected and are tested for instant-read cotinine (a metabolite of nicotine; COT One Step Cotinine Test Device; cut-off of 200 ng/ml), cannabinoids (11-nor-9-carboxy-Δ9-tetrahydrocannnabinol [THCCOOH], cut-off of 50 ng/ml), and other substances (amphetamines, opioids, etc.). Instant-read cotinine tests are used to inform inclusion, as well as determining abstinence at weeks 2–12 to deliver incentives. Collected urine samples are aliquoted and frozen at all sites to be analyzed by a clinical laboratory for quantitative cotinine (level of detection of 50 ng/ml) and cannabinoid levels (11-nor-9-carboxy-Δ9-tetrahydrocannnabinol; THCCOOH; level of detection of 50 ng/ml). Breath CO samples are also collected at all in-person visits using the Micro+™ pro Smokerlyzer® from coVita.

#### Self-report assessments

All participants complete a battery of tobacco assessments, and additional cannabis assessments are administered to participants who are co-using cannabis. To capture detailed self-report metrics of cannabis use, cannabis quantification procedures are conducted at screening to determine an individual-specific unit of cannabis use. For plant material-based methods, grams of cannabis are estimated using a surrogate substance [29, 30]. Participants weigh out the average amount of cannabis they use for each combustible modality endorsed (e.g., joints, blunts) and report the estimated dollar amount associated with that quantity (yields grams per method). That amount then serves as the individual’s standard unit for future reporting. This quantification procedure has been successfully implemented previously [31, 32] and has been shown to improve the predictive validity of relevant clinical outcomes [33]. For edibles and vaped cannabis, participants are asked about product type and to estimate the amount of cannabis in this method. For oils/concentrates, amount is based on the size of the cartridge, concentration of THC in the cartridge, and amount of hits to finish the cartridge. This is then used to calculate the grams of cannabis consumed. Timeline Follow-back (TLFB) [34, 35] is administered at screening, week 16, and week 26 to assess the frequency and quantity of tobacco use (cigarettes per day and other tobacco use), cannabis (using the standard units per method determined by quantification procedures), alcohol and other drug use.

#### Daily diaries

Using REDCap [28]/Twilio™ integration, a survey link is sent via text message to participants every morning to assess their substance use (e.g., cigarettes, other tobacco, cannabis, other substances) in the previous day. Surveys begin after screening and last until the week 12 EOT visit. Our research team has previously used daily diaries through REDCap [36, 37]. Based on methods of cannabis use endorsed, additional quantification questions are administered (number of “standard” joints, times used, hits taken, milligrams, etc. based on method of use). Missing data are collected at the next study visit using TLFB procedures.

#### Medication adherence

Medication adherence is tracked through several methods. First, participants receive Redcap/Twilio survey links via text message twice daily during the treatment period to record video of themselves taking study medication [37]. Second, pill counts are conducted weekly based on returned medication bottles. Third, smart caps are used with each medication bottle to time stamp each bottle opening.

### Data analytic plan

#### Propensity score methods

Non-randomized trials, like the current study, suffer from inherent selection bias due to systematic baseline differences in study populations [38]. The use of propensity scores (PS) has been shown to reduce selection bias and increase parameter estimate precision in non-random designs better than traditional covariate adjustment [39]. Augmented propensity score weighting strategies (AIPW) were chosen as the primary analytic approach as opposed to matching or stratification such that all study participants will be retained in the final analysis and statistical power would be preserved with the proposed sample size. In the current study’s population we expect age and race [40] to be imbalanced as well as baseline severity of tobacco use other relevant variables that may affect outcomes [11]. To account for such differences, augmented inverse probability of treatment weighting using the propensity score (AIPW) methods will be used to account for observed covariate values [41]. This methodology provides unbiased parameter estimates and standard errors of the average treatment effect even when either the propensity model or outcome model is misspecified [42]. All observed variables that potentially influence group membership will be included in the PS calculation (gender, education, motivation to quit, nicotine dependence, social influences, etc.). Prior to model analysis, weighted means of the measured baseline characteristics will be assessed between groups to assess balance. Additionally, weights will be checked for values close to either zero or one and adjustments to the probability model will be made when necessary.

#### Statistical analyses

To assess the difference in tobacco PPA at the week 12 EOT visit between groups (Aim 1), inverse probability weighted logistic regression models will be developed. The inverse of the probability of being in the cannabis co-use cohort will be used while adjusting for covariates [42]. To assess the association of baseline nicotine dependence with cannabis use rates during study treatment (co-use group only; Aim 2), we will use generalized linear mixed effects modeling with cannabis use rates and amounts during the final 4 weeks of treatment as the primary indicator of cannabis use severity and baseline nicotine dependence group as the primary model predictor. In addition to grouping by nicotine dependence severity, continuous levels of dependence will be modeled to test linear and quadratic relationships between nicotine dependence and cannabis use during the final 4 weeks of treatment. Rates of cannabis use will be assessed by dependence severity group over all weekly study visits to determine if those with increased severity exhibit a different cannabis use trajectory (slope and pattern). Finally, the relationship between baseline cannabis use and tobacco outcomes will be explored (Aim 3; exploratory aim). Generalized linear mixed effects models will be used with weekly 7-day PPA from tobacco as the primary model outcome. In addition to tobacco abstinence, weekly average cigarettes per day will be examined as an indicator of use reduction differences over time. Longitudinal patterns in tobacco abstinence and use amounts will be modeled through the inclusion of baseline cannabis use severity indicators, study visit, and baseline tobacco use rates as model covariates. Additionally, differential effects over time will be examined with the inclusion of the interaction of cannabis use severity and visit. Model-based means and associated standard errors will be used to test group level differences throughout treatment.

#### Power and sample size calculation

In an adolescent and young adult (ages 14–21) tobacco cessation trial conducted by our group evaluating the efficacy of varenicline [43], participants who did not co-use cannabis had double the odds of tobacco abstinence compared to participants who did co-use cannabis (26.0% vs. 12.2%; *p* = 0.021) [44]. Based on those results, we expected nearly double the abstinence rates among participants who were only using tobacco (compared to those co-using cannabis). Given previous studies on varenicline’s efficacy that have demonstrated rates of abstinence around 50% after 12 weeks of treatment [45, 46], we assumed a similar abstinence rate in tobacco-only controls (45%). We further assumed an abstinence rate of 20% among participants co-using cannabis (Δ = 25%), 80% power with a 5% type 1 error rate, and a 2:1 sampling ratio, all resulting in a sample size of 82 participants needed in the cannabis co-using cohort and 41 in the tobacco-only cohort. However, to account for the inclusion of multiple study sites, an additional fixed factor of site will be included in analytic models. Assuming the proportion of variance in groups due to study site and other covariates may be moderate (*R*^2^ = 0.25) and attrition will not exceed 25%, a randomized sample of 208 study participants is intended (139 participants co-using cannabis and 69 participants only using tobacco).

### Design decisions, challenges, and study modifications

#### Design decisions

*Age* The study sample is restricted to ages 18–40. While restricting the upper age limit reduces the pool of potential participants, it will also ensure the tobacco-only control group is similar to the cannabis co-use group in terms of age and potentially other characteristics that will affect the ability to compare groups. In a previous adult tobacco cessation trial conducted by our group [37], participants who co-used cannabis were similar to tobacco-only participants on several demographic and tobacco use characteristics but differed in age (M = 43.4; SD = 11.6 for tobacco-only; M = 34.7; SD = 8.7 for co-using participants). This is consistent with previous national data, suggesting cannabis use (and co-use) is most prevalent among 18–34 year-olds [47, 48], though cannabis-tobacco co-use rates continue to evolve and increase among other adult age groups [49].

*Excluding for blunt use* We considered excluding participants who use blunts. Blunts are hollowed out cigars/cigarillos filled with loose-leaf cannabis. Blunt use exposes participants to tobacco via the cigar/cigarillo wrapper [50] and may interfere with the ability to detect tobacco abstinence (via elevated cotinine levels). However, it was determined the inclusion of participants who use blunts as a cannabis method was essential given racial/ethnic differences in blunt use [51–53], the popularity of blunts among young adults [54], and excluding blunt users would limit generalizability. Blunt use, as it affects cotinine levels and the detection of tobacco abstinence, is discussed with participants as part of tobacco cessation counseling.

*Definition of co-use* There are no standard definitions of cannabis co-use and inclusion criteria were crafted to allow for a wide spectrum of cannabis severity. Participants must meet a minimum threshold of cannabis use to be considered in the co-using sample; however, this threshold still allows for variability in co-use patterns. Previous studies have categorized cannabis use based on national household survey recommendations [55–57], with 20 days of use in the past 30 indicating regular use, while intermittent use is defined as some past month use, but less than 20 days out of the past 30. Participants in this study must have used cannabis on at least 10 days out of the past 30 and/or must test positive on a qualitative urinary cannabinoid assay. Days of cannabis use in the past 30 will be collected through Timeline Follow-back (TLFB) procedures [35] at baseline, and while over- or under-reporting of cannabis use may occur, batched quantitative urinary cannabinoid assays will contribute to the appropriate categorization of severity.

#### Challenges

*Human subjects research restrictions* This study began enrollment in February 2020. Soon thereafter, in response to the COVID-19 pandemic and university guidance, procedures were modified to make use of remote study visits. A major challenge for this study has been the reliance on urine collection to capture cotinine and cannabinoids for primary and secondary outcomes. When possible, at-home urine collection is completed, and samples are frozen by participants and returned to study staff as soon as possible. When in-person visits resumed in the summer of 2020, we attempted to retain as many remote procedures as possible to encourage retention in the study and reduce missing data, though this does affect the ability to capture all biochemical measures of tobacco and cannabis use.

*Nicotine vaping exclusions* Regular use of other tobacco/nicotine products is exclusionary in this trial. Given the age range for inclusion (ages 18–40), poly-nicotine product use has been an ongoing challenge for enrollment. Among potential participants who contact study staff to learn about the study, 43% have endorsed other nicotine use in the past 30 days (mostly vaping). Research staff assess if interested callers are willing to abstain from other tobacco/nicotine products prior to screening and during the study, allowing for 2–4 weeks of reduced or no other tobacco use before enrollment. Nearly all callers are willing to abstain from other tobacco/nicotine, but this delay in scheduling a screening visit has resulted in many being lost prior to their visit.

*Study drug* Varenicline is the most efficacious pharmacotherapy for tobacco cessation [58], and as such, was selected as the study pharmacotherapy to be given to all participants. While other treatments exist for tobacco use disorder, the goal of this study is to achieve sufficient levels of tobacco abstinence to test hypotheses regarding the impact of cannabis co-use. Varenicline is also a first-line tobacco cessation pharmacotherapy, so its use in this trial mimics the clinical environment. Notably, recent data suggest varenicline may have an effect on cannabis use and may be a candidate treatment for CUD [59]. Data from our group’s youth tobacco treatment trial showed no evidence of varenicline reducing cannabis use among participants who also used tobacco [44], though cannabis use was not specifically addressed in that trial. Additionally, despite varenicline’s demonstrated safety [60, 61], there remain concerns regarding the use of this pharmacotherapy, particularly with co-occurring psychiatric disorders. That has remained a challenge in terms of interested callers not being willing to take varenicline or meeting exclusion criteria for safety. Further, availability of study drug was temporarily affected during the trial given the recall of Chantix® in July 2021.

## Conclusions

This study is the first prospective examination of the impact of cannabis use on tobacco cessation outcomes. Results will be uniquely positioned to inform the treatment of tobacco use among those co-using cannabis, which is an increasing proportion of the population [8, 49]. Future interventions for those who use both cannabis and tobacco may require enhanced and/or stepped care for tobacco cessation support based on cannabis use severity and may need to consider the potential of compensatory cannabis use during tobacco cessation. There may be individual differences in patterns of compensatory cannabis use that emerge based on co-use characteristics or the underlying relationship between substances that should be addressed during treatment. Interventions may also need to consider a lack of interest in cannabis cessation among those who use both products. As cannabis use rates continue to increase and the landscape of cannabis regulation changes, individuals who co-use both tobacco and cannabis will continue to be common in clinical trials and clinical care. Co-use may require tailored and potentially enhanced tobacco treatment and the results from this study will help to guide tobacco treatment among those who co-use cannabis and provide them with the best chances of successful long-term abstinence.

## Data Availability

Data will be made available upon written request to the corresponding author and with the execution of a data use agreement.
